# Insight into impact of sewage discharge on microbial dynamics and pathogenicity in river ecosystem

**DOI:** 10.1038/s41598-022-09579-x

**Published:** 2022-04-27

**Authors:** Yuyang Xie, Xiaolin Liu, Haiwei Wei, Xue Chen, Ningji Gong, Shakeel Ahmad, Taeho Lee, Sherif Ismail, Shou-Qing Ni

**Affiliations:** 1 Shandong Provincial Key Laboratory of Water Pollution Control and Resource Reuse, School of Environmental Science and Engineering, Shandong University, Qingdao, 266237 Shandong China; 2 Suzhou Research Institute, Shandong University, Suzhou, 215123 Jiangsu China; 3 Department of Emergency, The Second Hospital, Cheeloo College of Medicine, Shandong University, Jinan, 250033 Shandong China; 4 Department of Soil and Environmental Sciences, Muhammad Nawaz Shareef University of Agriculture, Multan, Pakistan; 5 Department of Civil and Environmental Engineering, Pusan National University, Pusan, 609-735 Republic of Korea; 6 Environmental Engineering Department, Zagazig University, Zagazig, 44519 Egypt

**Keywords:** Environmental impact, Microbial ecology

## Abstract

Direct sewage discharge could cause copious numbers of serious and irreversible harm to the environment. This study investigated the impacts of treated and raw sewage on the river ecosystem. Through our analysis, sewage carried various nutrients into the river, leading to changes in the microbial community in the river and reducing the diversity and richness of bacteria. The relative abundances of *Hydrogenophaga*, *Thauera*, *Planctomyces*, *Zoogloea*, and *Pseudomonas* boosted from 0.25, 0.01, 0.00, 0.05, and 0.08% to 3.33, 3.43, 0.02, 6.28, and 2.69%, before and after raw sewage discharge, respectively. The gene abundance of pathogenic bacteria significantly increased after raw sewage discharge. For instance, the gene abundance of *Vibrio*, *Helicobacter, Tuberculosis,* and *Staphylococcus* augmented from 4055, 3797, 13,545, 33 reads at Site-1 to 23,556, 13,163, 19,887, 734 reads at Site-2, respectively. In addition, according to the redundancy analysis (RDA), the infectious pathogens were positively related to the environmental parameters, in which COD showed the highest positive correlation with *Mycobacterium tuberculosis*. Additionally, river self-purification may contribute to improving water quality and reducing pathogenicity. The outcomes of this study showed that direct discharge brought pathogens and changed microbial community structure of the river.

## Introduction

With the massive worldwide population increase, water has been predicted to become one of the scarcest resources in the twenty-first century. Moreover, the UN’s World Water Development Report, 2017, said globally 80% of sewage (> 95% in developing countries) is directly released to the environment. Numerous questions have been raised about the ability of wastewater treatment programs to remove pathogens from wastewater, in which many waterborne diseases are associated with supposedly treated water supplies. Untreated wastewater can threaten the human and ecological systems by enriching pathogenic bacteria^1^. Moreover, a body of literature has confirmed the presence of severe acute respiratory syndrome coronavirus 2 (SARS-CoV-2) in wastewater^2–4^. SARS-CoV-2 possibly spreads through the wastewater treatment network^5^. When raw sewage contaminates the aquatic environment, these pathogens can be transmitted through the water to those who use the water for swimming, boating, and fishing, causing a range of potential risks^6^. However, there are many ways for untreated wastewater to enter rivers. When sewage treatment cannot treat too much wastewater or when it is raining heavily, raw sewage will be discharged directly into river channels, polluting the environment and making the water quality worse. Natural disasters, such as earthquakes and floods, also make raw sewage flow into rivers, transporting pathogens into them^7^. Once sewage is delivered to receiving waters, a series of environmental changes will occur. Over time, river dilution, storage in sediments, and the intrinsic characteristics of the microorganisms and other related processes may alter the destiny of the bacteria and pathogens of concern.

The direct discharge of sanitary wastewater may cause environmental diversity changes. Sewage discharge without proper treatment significantly altered the concentrations of different organic and inorganic contaminants in the receiving water bodies^8^, especially ammonia (NH_4_^+^), nitrate (NO_3_^−^), total phosphorus (TP), and chemical oxygen demand (COD). The increase of these contaminants could trigger the aquatic organism by causing eutrophication of the catchment^9^. David et al. found that the high NH_4_^+^ concentration negatively influenced the functional performance and taxonomic richness of the microbial community of 10 different WWTPs located across Switzerland^10^. Moreover, phosphorus content was positively correlated with the microbial biomass^11^ and occupied an important position among environmental factors that affect the microbial community of sediments^12,13^.

Several common infectious pathogens, such as *Vibrio cholera, Staphylococcus aureus, Mycobacterium tuberculosis*, and *Helicobacter pylori*, exist in aquatic environments. *Vibrio cholerae,* a well-known internationally quarantinable infectious pathogenic bacterium, could cause human enteric infection^14^. *Vibrio cholera* O1 is widely distributed in aquatic environments, namely rivers, ponds, sewage, and estuaries, in many developing countries such as Haiti^15^. Reidl et al. isolated *Vibrio cholerae* from estuarine and aquatic environments^14^. *Staphylococcus aureus* may cause local purulent infection, pneumonia, and colitis after enrichment in water^16^. Zieliński et al. found sewage treatment plants are a sizeable source of drug-resistant staphylococci harboring virulence genes^17^. Moreover, *Mycobacterium tuberculosis* normally enters the host via mucosal surfaces, usually through the lung after inhaling infectious droplets from an infected individual and occasionally via the intestine after ingesting infected material^18^. Velayati et al. isolated *Mycobacterium tuberculosis* from water samples collected in Tehran, Iran metropolitan area^19^. Many diseases, such as gastritis, peptic ulcer, and lymphoid proliferative gastric lymphoma, are caused by *Helicobacter pylori* infection^20^. West et al. reported the capabilities of *Helicobacter pylori* to survive in various buffers at room temperature over a range of physical variables, which means that *Helicobacter pylori* may survive in a natural aquatic environment^21^. Therefore, pathogenic bacteria in the water environment will have direct or indirect effects on human beings or animals. The study of their microbial community composition and influencing factors is urgent for future treatment measures.

The lack of a thorough understanding of the survival and persistence of different microbial types in different conditions and environments is one of the major gaps in the knowledge of pathogenic microorganisms in wastewater. Previous studies focused on the abundance of pathogenic bacteria and the relationship between the contaminants and pathogenic bacteria in rivers contaminated by raw sewage discharge^22,23^. However, it is unclear whether the source of pathogenic bacteria in the river comes from sewage itself or whether it is caused by the environmental change after sewage discharge. Also, a deep insight into microbial and genetic responses to river self-purification needs further investigation. Thus, the main target of the present study is to fill in this knowledge gap by investigating the environmental parameters and the microbial community structures of different sites along the Daxin River, representing treated and raw sewage discharge points. To achieve this objective, four sample sites were selected before and after treated and untreated sewage discharge points. The microbial community composition was determined by high-throughput 16S rDNA amplicon sequencing. Also, the components and concentrations of pathogenic bacteria were identified by qPCR and forecasted by PICRUSt analysis. The concentration of environmental factors, including ammonia, nitrite, nitrate, phosphate, COD, and temperature of the sediment-overlying water, were analyzed. Moreover, redundancy analysis (RDA) was employed to explore the relationship between environmental factors and microbial community structure. The specific objectives of this study are to (1) provide more information about the water quality and microbial community structure before and after domestic wastewater pollution, (2) explore the composition of the pathogenic bacteria community in the Daxin River, and (3) evaluate the relationship of infectious pathogens and functional bacteria to different environmental factors.

## Materials and methods

### Description of Daxin River and sample collection

**Figure 1:**
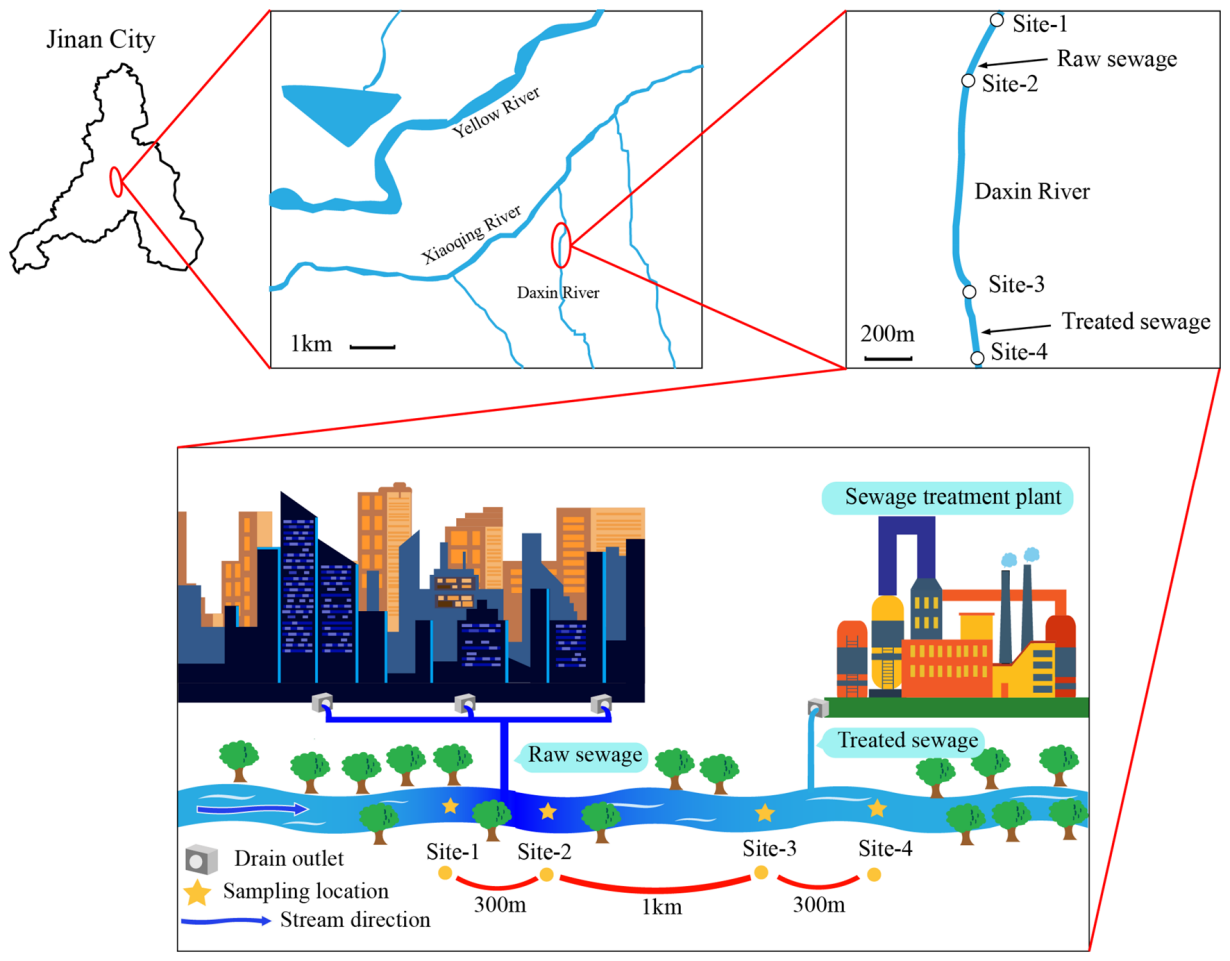
Daxin River sampling points locations.

Jinan is located in northwest Shandong Province, China, near the confluence of two important water systems, the Yellow River and the Xiaoqing River. The Daxin River is a tributary of the Xiaoqing River. Sediment and sediment-overlying water samples were harvested from four different sites along the Daxin River. The four sites were designated, from upstream towards downstream of the river, as Site-1 (117°6′26″N, 36°42′35″E), Site-2 (117°6′24″N, 36°42′44″E), Site-3 (117°6′26″N, 36°44′1″E), and Site-4 (117°6′22″N, 36°44′17″E), representing the discharge points of treated and raw sewage (Fig. 1). The raw sewage was discharged between sites 1 and 2; therefore, Site-2 was directly polluted by raw sewage. The sewage discharged from the drain outlet was domestic wastewater from the nearby neighborhood. Site-3 was located about 3 km downstream of Site-2. A sewage treatment plant (STP) discharge port is located between Site-3 and Site-4. The water samples at each site were from the overlying water of the river bed sediments and were collected in Niskin bottles and filtered through 0.22-μm syringe filters for further analysis. Water quality parameters were measured immediately at sample sites. A Peterson mud sampler was used to collect the surface sediment samples by harvesting 0–10 cm of sediment depth. Sediment samples were frozen at − 80 °C for subsequent analysis.

### Analytical methods

The concentrations of NO_3_^–^N, NO_2_^–^N, and NH_4_^+^-N were measured according to the standard method (TU1810-PC, Purkinje General, China)^24^. The sediment-overlying water temperature was measured by a centigrade thermometer at each site. The pH was measured using a pH meter (PhS-3C, Rex Electric Chemical, China) during water sampling. The content of total phosphorus (TP) was determined by the colorimetric method of vanadium-molybdenum phosphoric acid. The measurement of a COD was carried out by COD rapid measuring instrument (MI-80K, China).

### DNA extraction and quantitative real-time PCR (q-PCR) analysis

The Power Soil™ DNA Isolation Kit (Takara Company) was used to extract the total DNA of the freeze-dried sediment samples^25^. The qPCR (Roche Light Cycler 480, Switzerland) was used to detect the gene concentration of *Vibrio cholerae, Staphylococcus aureus, Mycobacterium tuberculosis,* and *Helicobacter pylori*. The primer sequences of the genes of *Vibrio cholerae* were GCCAAAATTGTGCGTATCAG and ATAATCTTGGGCAATCGCA. The primer sequences of the genes of *Staphylococcus aureus* were CTAATACTGAAAGTGAGAAACGTA and TCCTGCACAATCGTACTAAA. The primer sequences of the genes of *Mycobacterium tuberculosis* were CCTGCGAGCGTAGCGTCGG and CTCGTCCAGCGCCGCTTCGG. The primer sequence of the genes of *Helicobacter pylori* were GCCAATGGTAAATTAGTT and CTCCTTAATTGTTTTTAC. The qPCR reaction mixture (20 μL) included 2× SYBR Green PCR master mix (10 μL), forward primer (0.4 μL), reverse primer (0.4 μL), template DNA (1 μL), and dd H_2_O (8.2 μL). The primers used to identify the infectious pathogens were designed according to the conservative sequence of characteristic genes. The annealing temperature of qPCR was predicted using the software primer5.0 based on its “GC” ratio in the whole sequence^26^. The qPCR data were analyzed using Abs Quant/2-order derivatives of Max software^27^.

### High-throughput 16S rDNA amplicon sequencing

The next-generation sequencing was applied to determine the microbial community structure based on the universal primer pair of 16S rDNA^28^. The V4 amplicons were sequenced using primers 515F (GTGCCAGCMGCCGCGGTA) and 806R (GGACTACNVGGGTWTCTAAT) and the pair-ended method on the Illumina Miseq sequencing platform at Personal Biotechnology Co., Ltd.^29^. PICRUSt was used to predict the potential of a sample using 16S rDNA amplicon sequencing. KEGG Orthology (KO) was used to classify all genes that are homologous to a specific gene whose function is known to be in the same category^16^.

### Laboratory incubation experiments for the growth potential of typical bacteria

To characterize the environmental media affecting microbial growth in Daxin River ecosystems, typical Gram-positive and negative bacteria, *Staphylococcus aureus* and *Escherichia coli*, were selected to investigate the effects of river water and sediment on microbial growth at different pollution levels. Unpolluted river water, Unpolluted sediment, polluted river water, polluted sediment, recovered river water, and recovered sediment samples were collected and made as culture media for the growth of the two bacteria, and the biomass-time curves of the bacteria were examined to determine and verify the dominated environmental media that promote bacterial growth in rivers. The water samples from Site-1, Site-2, and Site-4 were collected and filtered using 0.22 μm polyethersulfone membrane to make as unpolluted water culture medium, polluted water culture medium, and recovered water culture medium. The sediment samples from Site-1, Site-2, and Site-4 were centrifuged at 1000*g* for 5 min. The supernatant was decanted and the centrifuged sediment was retained. Then, 10 mL deionized water was added to the sediment, and the sediment was sonicated for 20 min to extract the nutrients from the sediment, followed by centrifugation at 1000*g* for 5 min. The supernatant was extracted and filtered using 0.22 μm polyethersulfone membrane to make as unpolluted sediment culture medium, polluted sediment culture medium, and recovered sediment culture medium. All culture media were sterilized by autoclaving at 121 °C for 15 min. The growth curves of both bacteria were examined using an automated microbiology growth curve analysis system (Bioscreen FP-1100-C, Finland). The volume of culture media was 1 mL, including 1% of bacterial seed solution (*Staphylococcus aureus* and *Escherichia coli,* OD_600 nm_ ≈ 1). The temperature was set at 30 °C and the value of OD_600 nm_ was recorded every 60 min.

### Data analysis

The community richness was estimated by the Chao1 and community diversity was estimated by Simpson and Shannon indices using the MOTHUR software^30^. CANOCO version 4.5 software was used for RDA to evaluate the relationship between the environmental parameters and microbes.

## Results

### Water quality and environmental parameters of the sampling sites

The sediment-overlying water quality parameters of the four sites were summarized in Table 1. COD and NH_4_^+^-N concentrations significantly increased from 47.3 and 25.6 mg/L to 74.3 and 73.3 mg/L, respectively, after the raw sewage was directly discharged into the river. Likewise, NO_3_^–^N and TP concentrations slightly increased from 0.2 and 1.6 mg/L at Site-1 to 1.2 and 5.7 mg/L at Site-2, respectively. However, due to river self-purification that occurred between Site-2 and Site-3, all sediment-overlying water quality parameters returned to their average level before the discharge of raw sewage, except for COD which witnessed a slight decrease from 74.2 to 62.8 mg/L at Site-3. In contrast, there were relatively small changes after the discharge of sewage treatment plant effluent to the river. COD slightly decreased from 62.8 to 52.3 mg/L, while NO_3_^–^N increased from 0.23 to 12.2 mg/L at Site-3 and 4, respectively.

**Table 1:** Water quality and environmental parameters of the four sites.

	Site-1	Site-2	Site-3	Site-4
pH	7.8 ± 0.5	7.5 ± 0.7	8.3 ± 0.9	7.8 ± 0.5
COD (mg/L)	47.3 ± 3.2	74.3 ± 4.2	62.8 ± 5.1	53.2 ± 3.2
NH_4_^+^-N (mg/L)	25.6 ± 1.5	73.8 ± 5.2	25.6 ± 2.1	8.7 ± 1.1
NO_3_^–^N (mg/L)	0.2 ± 0.06	1.2 ± 0.1	0.2 ± 0.05	12.2 ± 1.5
NO_2_^–^N (mg/L)	0.2 ± 0.04	0.1 ± 0.01	0.7 ± 0.06	0.1 ± 0.03
T (^o^C)	28.1 ± 4.0	28.7 ± 2.1	29.7 ± 1.5	32.7 ± 2.3
TP (mg/L)	1.6 ± 0.1	5.7 ± 0.8	1.2 ± 0.3	0.3 ± 0.1

### Analysis of the community composition

The microbial community structure was determined by high-throughput 16S rDNA amplicon sequencing. The total obtained operational taxonomy units (OTUs) were 28,427, 28,354, 32,810, and 39,105 for the four sites, respectively. The Chao1, Simpson, and Shannon indexes were calculated to determine the richness and diversity of the microbial community^31^. As shown in Table 2, The Chao1, Simpson, and Shannon indexes of sediment samples at Site-1 decreased from 874, 0.955, and 6.771 to 869, 0.953, and 6.484 at Site-2, indicating the richness and diversity of the bacterial community were negatively influenced by untreated sewage discharge. Furthermore, the values of Chao1 kept increasing from Site-2 to Site-4 indicating the increase of the richness of the microbial community along with river self-purification.

**Table 2:** Analysis of microbial richness and diversity.

Sample	Reads	Chao1	Simpson	Shannon
Site-1	28,427	874	0.95497	6.7710
Site-2	28,354	869	0.95311	6.4841
Site-3	32,810	1535	0.99000	9.2100
Site-4	39,105	2577	0.99000	9.1100

Chao1 indicates richness, Shannon and Simpson indicate diversity.

**Figure 2:**
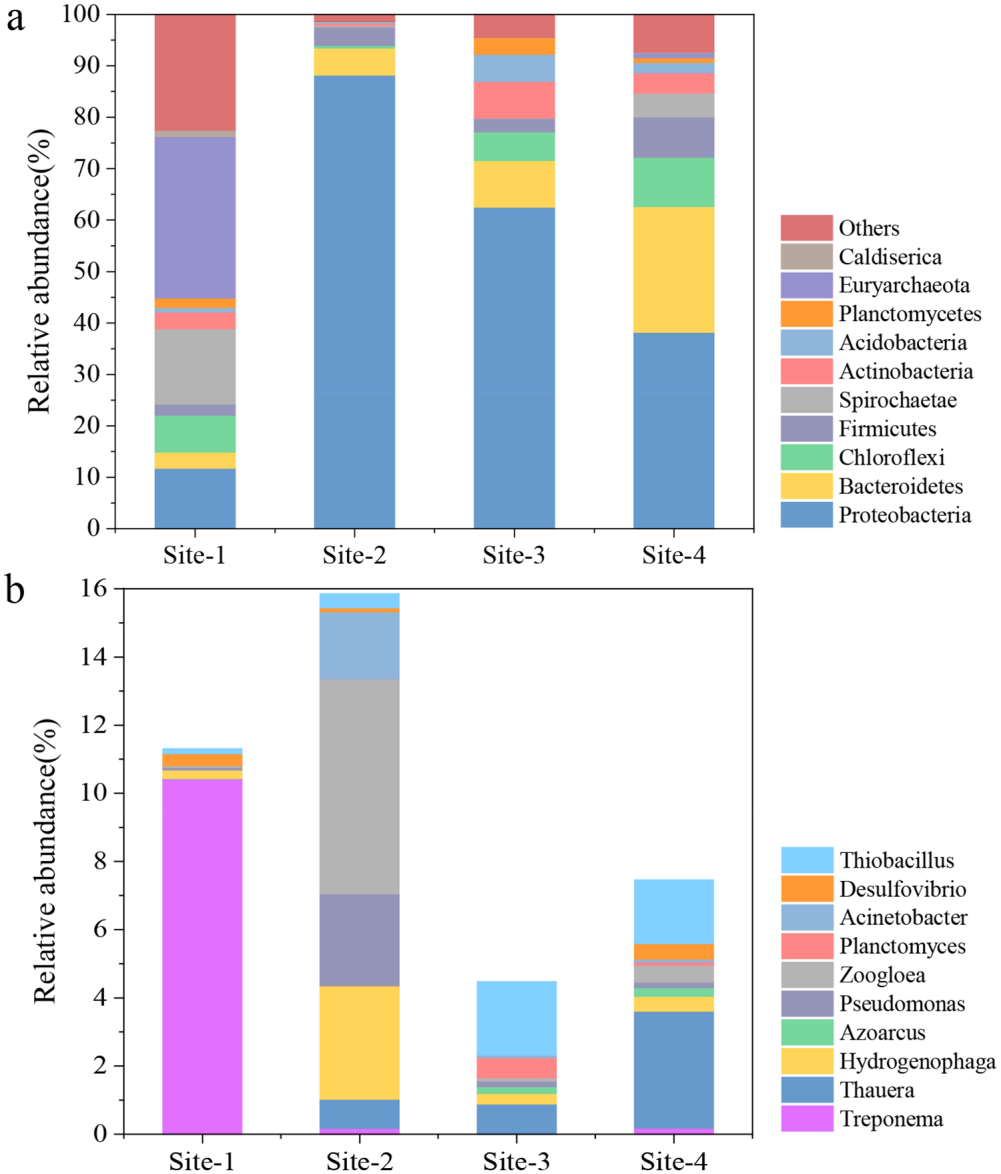
Microbial composition in four sites at the phylum (**a**) and genus (**b**) levels.

Fifty-nine phyla were detected from the four sites, and ten predominant phyla were shown in Fig. 2a. Relative abundance of *Proteobacteria* increased from 11.8% at Site-1 to 88.2% at Site-2 and then gradually decreased to 62.54% at Site-3 and 38.2% at Site-4. Conversely, relative abundance of *Chloroflexi* decreased from 7.18% (Site-1) to 0.53% (Site-2) and then gradually recovered to 5.52% (Site-3) and then 9.58% (Site-4); *Spirochaetae* decreased from 14.67% (Site-1) to 0.3% (Site-2) and then gradually recovered to 4.68% (Site-4); *Euryarchaeota* decreased from 31.37% (Site-1) to 0.07% (Site-2), however, they only recovered to 0.91% (Site-4). Additionally, *Actinobacteria*, *Acidobacteria*, *Planctomycetes*, and *Caldiserica* were reduced slightly to 0.41, 0.32, 0.05, 0.01% at site-2, respectively, and gradually recovered to 3.94, 2.02, 0.94, 0.15% at site-4, respectively. The ten dominant genera were shown in Fig. 2b. These genera are mainly related to nitrogen metabolism, COD degradation, phosphorous removal, and sulfur removal. *Treponema*, which can convert NO_2_^−^ to NO_3_^−^, had the highest abundance percentage of 10.42% at Site-1. Two denitrifying bacteria were enriched at Site-2, i.e., *Hydrogenophaga* (3.33%) and *Thauera* (0.86%)*. Thauera*, a denitrifying related bacteria producing N_2_ using NO_2_^−^ or NO_3_^−^ as the electron donor^32^, was also enriched at Site-4. *Azoarcus* that performs nitrogen fixation^33^ appeared at Site-2 then kept growing in Site-3 and Site-4. The *Zoogloea* and *Pseudomonas* genera whose main function is to degrade organic matters increased sharply at Site-2. The *Pseudomonas* genus, responsible for the removal of phosphorus, was also dominant in Site-2 coupled with *Acinetobacter*. Their enrichment may be due to the high phosphorus content in the raw sewage, as evidenced by the highest TP content at Site-2 (Table 1). *Thiobacillus* and *Desulfovibrio* are related to sulfur removal, and *Thiobacillus* was enriched at Site-3 and Site-4. Furthermore, four important functional microbes were extracted and classified as denitrifying bacteria (DNB), anammox bacteria, organic degrading bacteria (ODB), and phosphorus-accumulating bacteria (PAOs) as shown in Table 3. Three genera of DNB were detected in almost all samples. In particular, the relative abundance of *Hydrogenophaga* at Site-2 (3.333%) was more than 13-fold of that at Site-1 (0.253%); however, it suddenly decreased at Site-3 and Site-4. *Azoarcus* was absent at Site-1 and appeared at Site-2 (0.005%) then continued to grow at Site-3 (0.194%) and Site-4 (0.244%). *Thauera* also showed an increasing trend from Site-1 to Site-4. Anammox bacteria *Planctomyces* were also detected at the genus level, and its highest abundance was 0.59% at Site-3. Organic degrading bacteria *Zoogloea* increased significantly to 6.28% at Site-2 and decreased to 0.093% and 0.497% at Site-3 and Site-4, respectively. PAOs bacteria include *Pseudomonas, Arthobacter, Nocardia, Beyerinkia, Ozotobacter, Aeromonas, Microlunatus,* and *Rhodocyclus*^25^. Only one PAOs genus was detected in all sites. The abundance of PAOs was much higher at Site-2 than that at Site-1, with a relative abundance of 2.69% and 0.08%, respectively. The relative abundance of all the above-mentioned functional bacteria at Site-2 is higher than those at Site-1, while only four bacteria related to COD and nitrate removal showed a continuous increase between Site-3 and Site-4.

**Table 3:** Percentage of the functional microbes in the four sites (%) at the genus level.

Type of bacteria	Name of bacteria	Site-1	Site-2	Site-3	Site-4
DNB	*Hydrogenophaga*	0.253	3.333	0.315	0.438
	*Thauera*	0.012	0.856	0.888	3.429
	*Azoarcus*	0	0.005	0.194	0.244
Anammox	*Planctomyces*	0	0.016	0.592	0.097
ODB	*Zoogloea*	0.047	6.28	0.093	0.497
PAOs	*Pseudomonas*	0.077	2.685	0.167	0.166

DNB, denitrifying bacteria; ODB, organic degrading bacteria; PAOs; phosphorus-accumulating bacteria.

### Pathogenic gene abundance based on PICRUSt prediction and qPCR results

**Figure 3:**
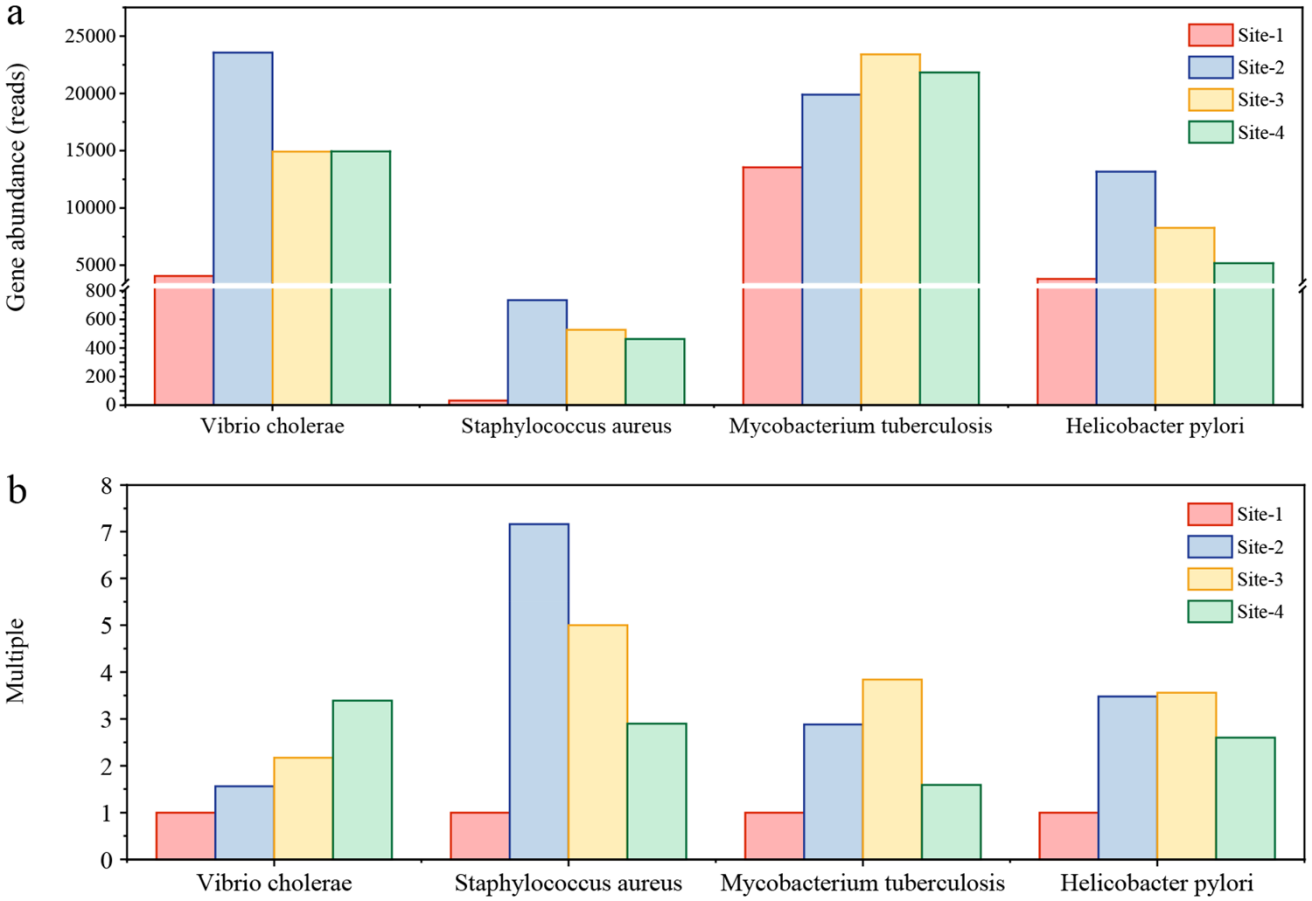
Prediction of abundance of genes related to infectious diseases (**a**) and the multiple relationship among them by qPCR (**b**) at four sites.

Pathogenic bacteria in water affect human and animal health. Pathogens could live in untreated wastewater for longer periods and spread further through water bodies^34^. Figure 3 shows the gene abundance of infectious diseases at four sites according to the forecast of the KEGG database (Fig. 3a) and the relationship among four sites based on qPCR results (Fig. 3b). There were significant differences in infectious diseases between Site-1 and Site-2, while no obvious variation was observed between Site-3 and Site-4. After being polluted by raw sewage, the gene abundance of infectious diseases increased sharply. The increased gene was related to *Vibrio cholera* pathogenic cycle process, whose gene amount increased from 4055 to 23,556 reads. The gene abundance of *Helicobacter pylori infection* increased from 3797 to 13,163 reads. The gene abundance of *Tuberculosis* also increased from 13,545 to 19,887 reads. The four kinds of pathogenic genes had the most remarkable variation among all the genes related to infectious diseases between Site-1 and Site-2. On the other hand, there is no remarkable big gap between the gene abundance of samples at Site-3 and Site-4. The values of these four infectious disease abundances were verified by qPCR, as shown in Fig. 3b. Compared to Site-1, the abundance of these four infectious diseases in Site-2, Site-3, and Site-4 increased. The abundance of *Staphylococcus aureus* at Site-2 far exceeded that of Site-1. The abundance of these four infectious diseases decreased at Site-4, compared to Site-3.

### The relationship of microbial structure to sediment-overlying water quality parameters

**Figure 4:**
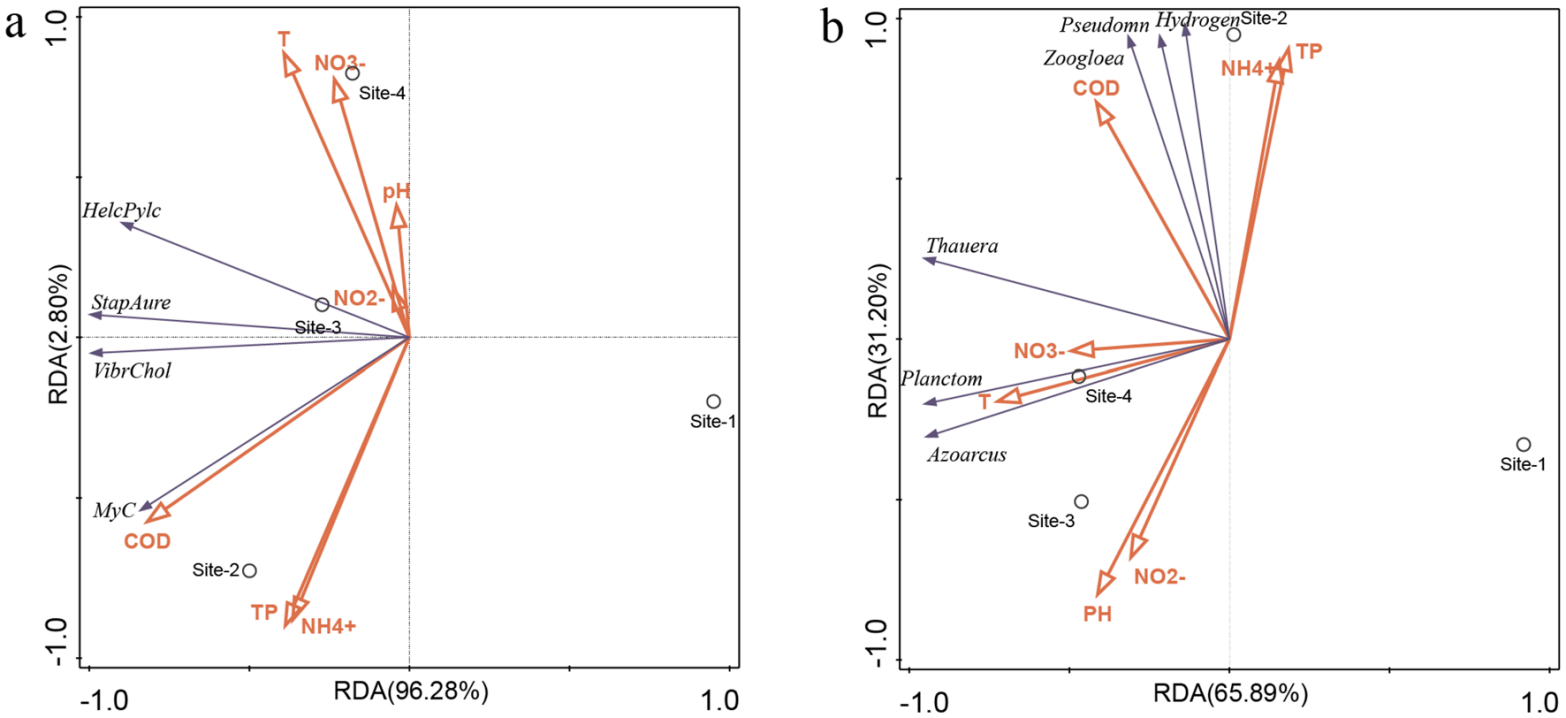
Redundancy analysis (RDA) of water quality parameters at the four sampling sites and their relationship to the microbial community structure of infectious pathogens (**a**) and functional bacteria (**b**).

The relationship between microbes and environmental variables was evaluated by redundancy analysis (RDA) (Fig. 4). The data used in Fig. 4a was from the abundance of genes related to infectious diseases in Fig. 3a and the environmental quality data, including COD, T, pH, NO_2_^−^, NH_4_^+^, NO_3_^−^, TP in Table 1. The data used in Fig. 4b was from the environmental quality in Table 1 and the relative abundance of functional bacteria in Table 3. Based on Fig. 4, there are significant differences between Site-1 and the other three sites. The seven water quality parameters have diverse behaviors in affecting the distribution of the infectious pathogens in the four sites. Site-2 has higher COD, TP, and NH_4_^+^ concentrations than other locations. Meanwhile, TP and NH_4_^+^ got a strong positive correlation with each other among all the environmental factors and they contributed to a large proportion in affecting the abundance of *Mycobacterium tuberculosis* (Fig. 4a). COD had less correlation to other parameters but strongly influenced the abundance of *Mycobacterium tuberculosis.* In addition, according to the results of RDA, the temperature at Site-4 was closely correlated to the growth of functional bacteria (Fig. 4b). Few functional bacteria were located in Site-1, while the discharge of raw sewage led to the most abundant and multiple distributions of functional bacteria at Site-2 (Fig. 4b). Many bacterial species at Site-2 were affected by COD, NH_4_^+^, and TP. Moreover, NO_3_^−^ and temperature contributed to the microbial community diversity at Site-3 and Site-4. The functional microbes at Site-3 were influenced by pH and NO_2_^−^_._

### Effect of various aquatic media on the growth of typical bacteria

**Figure 5:**
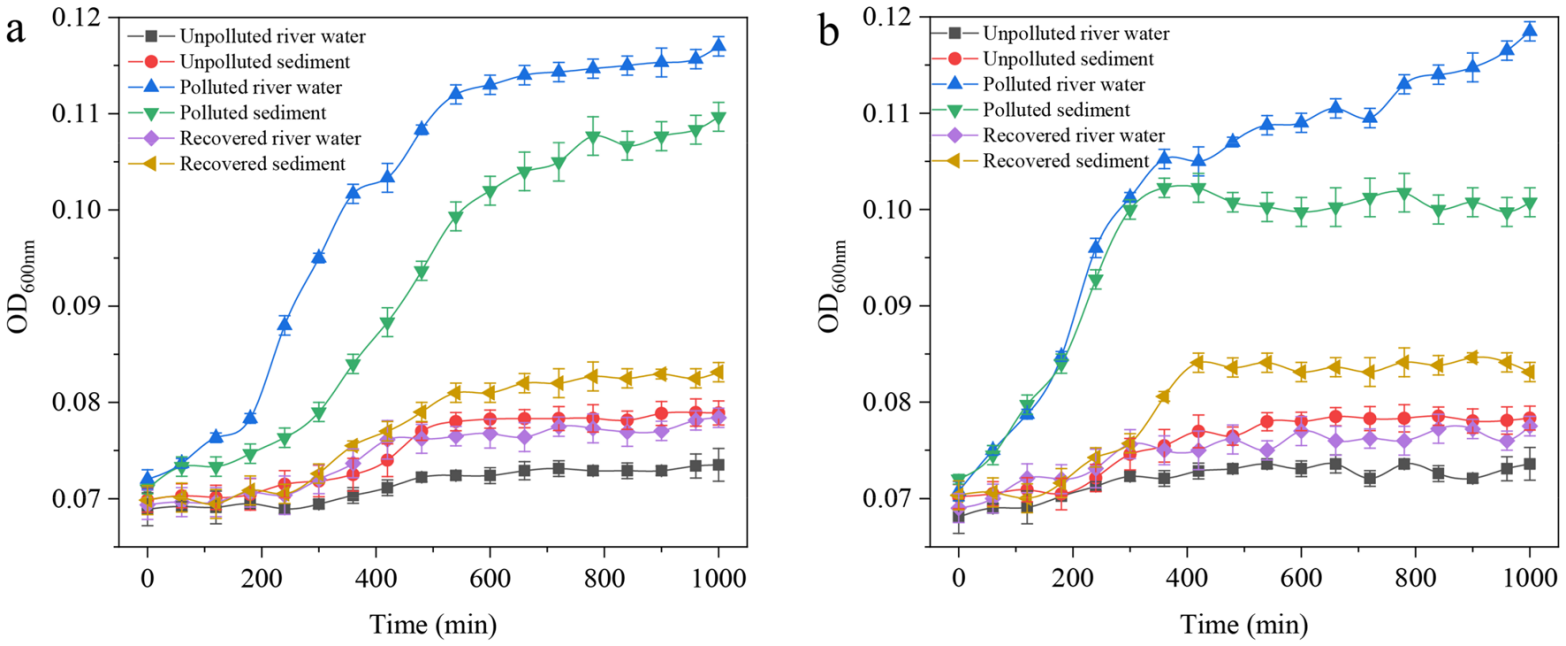
Growth of *Staphylococcus aureus* (**a**) and *Escherichia coli* (**b**) in different culture media.

This survey suggested that pathogenic bacteria could survive and multiply after sewage discharge. To investigate and verify the effect of various aquatic media on the growth of microbes, typical Gram-positive and negative bacteria, *Staphylococcus aureus,* and *Escherichia coli*, were selected to examine their growth potential in different media. Figure 5 showed the growth curves of *Staphylococcus aureus* (a) and *Escherichia coli* (b) in media from different sampling sites. Under the exposure of unpolluted water in Site-1, the bacterial densities of *Staphylococcus aureus* and *Escherichia coli* were almost unchanged and the value of OD_600 nm_ was maintained around 0.073. The unpolluted sediment still contained various nutrients, which slightly promoted the growth of *Staphylococcus aureus* and *Escherichia coli*, with OD_600 nm_ values reaching 0.079 and 0.078 at 1000 min, respectively. The polluted river water and sediment collected at Site-2, however, allowed the rapid growth of *Staphylococcus aureus* and *Escherichia coli*, with the OD_600 nm_ values reaching 0.120 and 0.117 at 1000 min, respectively. While the recovered river water obtained at Site-4 also promoted the growth of these two bacteria slightly, with both values of OD_600 nm_ reaching about 0.078 at the 1000 min. Compare with the recovered river water, the recovered sediment promotion was even more effective, with values of OD_600 nm_ reaching 0.082 and 0.084 at 1000 min. These results suggest that the polluted river water and the sediment contain nutrients to support the growth of *Staphylococcus aureus* and *Escherichia coli* at ambient temperature (30 °C).

**Figure 6:**
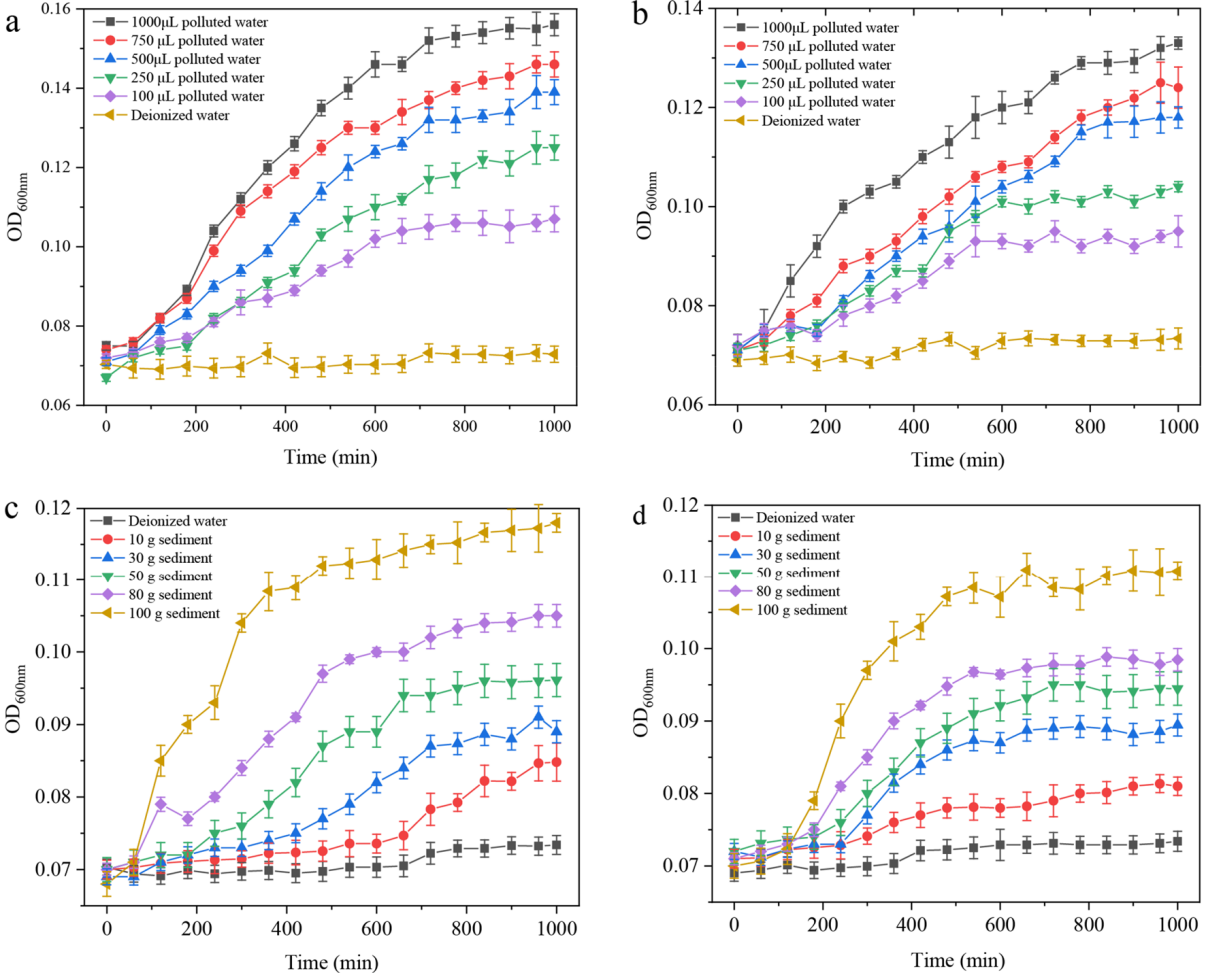
Growth of *Staphylococcus aureus* and *Escherichia coli* at different polluted river water concentrations (**a**,**b**) and different sediment mass additions (**c**,**d**).

To further verify the effect of polluted water and sediment in the river on pathogenic bacteria, growth experiments on two typical strains were conducted with different polluted river concentrations and different sediment mass addition. First, culture media containing different volumes of contaminated river water was made up to 1 mL with sterile water. Under the exposure of different concentrations of polluted water, the growth curves of *Staphylococcus aureus* and *Escherichia coli* were tested respectively, as shown in Fig. 6a,b. The higher the concentration of polluted water, the better the growth promotion effect of both strains. Second, to study the effect of sediment in Daxin River on the growth of pathogenic bacteria, the supernatant obtained from different sediment masses was used as culture medium to incubate these two strains of bacteria, as shown in Fig. 6c,d. Although the sediment supernatant was less effective than contaminated river water, there was still some promotion of both strains. The promotion on the growth of *Staphylococcus aureus* and *Escherichia coli* became more effective as the mass addition of sediment increased.

## Discussion

### The directly discharged sewage altered the microbial community structure

According to our analysis, the raw sewage altered the environmental conditions of the river, caused changes in the microbial community, and eventually led to a decrease in species richness and diversity. It may be due to the discharged treated sewage, containing high levels of nutrients, boosted the abundance of bacteria that thrive in eutrophic environments, and inhibited the growth of other bacteria. As pollutant concentrations decreased and water quality improved, the species richness increased again during the process from Site-3 to Site-4. Moreover, the abundance of pathogens, which belong to *Proteobacteria,* witnessed a noticeable growth after raw wastewater flowed into the river, as shown in Fig. 2. The taxonomic analysis found that *Proteobacteria*, *Bacteroidetes*, and *Firmicutes* were the most abundant phyla in all samples. *Proteobacteria* include various pathogens, such as *Escherichia, Salmonella, Vibrio,* and *Helicobacter,* and many bacterial species responsible for nitrogen transformation and had a positive correlation with NH_4_^+^ concentration^35^. The high relative abundance of *Proteobacteria* at Site-2 could be due to the increase in NH_4_^+^-N concentration from the direct discharge of sewage to the river; the high ammonia provided an appropriate living environment for *Proteobacteria* growth^35,36^. In a suitable environment, *Proteobacteria* competes with other native microorganisms and reduces microbial diversity^37,38^. With the gradual decrease of NH_4_^+^-N concentration at Site-3 and Site-4, the abundance of *Proteobacteria* gradually declined to 62.5 and 38.2%. Table 3 shows that the most significant change between Site-1 and Site-2 among the functional bacteria was *Pseudomonas,* which can remove low concentrations of TP from wastewater^39^. The discharge of raw sewage between Site-1 and Site-2 increased COD and nitrate contents, which provided sustainable and effective electron donors for denitrification, promoting the growth of denitrifying bacteria, such as *Hydrogenophaga and Thauera*. *Zoogloea* has been reported with high versatile metabolism capabilities such as nitrogen fixation, secretion of extracellular polymeric substance (EPS), and COD removal^40^. The increase of ammonia nitrogen content at Site-2 may be related to the high *Zoogloea* abundance at this site. The contents of *Zoogloea* increased at Site-2 after the raw wastewater surged in and then decreased in the following two downstream sites. RDA results suggested that functional bacteria, such as *Zooloea, Pseudomonas,* and *Hydrogenophaga,* were closely related to COD, NH_4_^+^, and TP. The DNB, which can restore NO_2_^−^, was enriched sharply at Site-2. The possible reason may be that NO_2_^−^, and NH_4_^+^ provide nutrition for DNB^41^.

### Raw sewage increased the pathogens content in the river and pathogenic bacteria could come from raw sewage directly and environmental changes

The discharge of pollutants from industrial processes causes adverse environmental impacts on aquatic ecosystems, such as eutrophication of water bodies, the introduction of pathogens, re-assembling of the microbial community structure in rivers^42,43^. This paper revealed that raw sewage remarkably contributed to the enrichment of pathogens in sediment samples of the river. From the results of PICRUSt prediction, the gene contents of four infectious diseases indeed increased after raw wastewater was discharged into the river, while most gene contents decreased after the discharge of treated wastewater. Further analysis proved that the PICRUSt prediction was in accord with the qPCR results, similar to a study conducted by Langille et al.^44^. In general, the discharge of raw sewage into rivers changed the water quality of rivers and caused the growth of pathogenic bacteria in these rivers. The abundance of pathogenic genes of Site-2 showed a significant increase, which was higher than the other three sites. With the purification of the river, the water quality gradually changed and the abundance of pathogenic genes decreased in Site-3. When the treated wastewater was discharged into the river, the pathogenic genes of site-4 did not change dramatically. Moreover, in the case of sediments being disturbed, it is likely that microorganisms, including pathogens, would remigrate into the sediment-overlying water column. Medema et al. reported that protozoa can settle from the water column into the sediment and, due to their size, remain undisturbed for long periods^45^. Thus, re-suspension events increase the potential risk potential to human health for those who are involved in activities in the aquatic environment. A suitable temperature is also an essential factor for the growth of pathogenic bacteria. When the pathogen is sown in pasteurized sewage, it can be infectious for up to 7 days, in deionized water at 25 °C for 22 days, and in these media for up to 4 weeks or more at lower temperatures (< 4 °C)^34^. *Vibrio* pathogens could be easy to remain active during treatment in rural sewage treatment plants and appear to be more abundant over a wide temperature range of 17.1 to 27.2 °C^46^. Furthermore, it was reported that the relatively high temperature in the range of 5–30 °C was beneficial to the growth of *Vibrio cholera*^47^. These demonstrate the temperature tolerance of these bacteria in water body. Therefore, the suitable temperature in Site-2 also contributed to the survival and maintenance of the pathogens. In addition, with the change of sediment-overlying water quality, mostly COD, in the river from Site-2 to Site-3, it was more suitable for *Mycobacterium tuberculosis* to be enriched than other bacteria.

Through the microbial community structure analysis, the microbial diversity at Site-2 decreased while the abundance of pathogenic bacteria increased. Here, the relationship of microbes and environmental factors was studied through incubation experiments for the growth potential of typical bacteria. Cui et al. found that the enteric pathogens in the urban rivers were likely to have originated from domestic sewage^48^. Suzuki et al. found *E. coli* grew in river water when sterilized sediment was supplemented^49^. Based on our results, pathogens in the Daxin River could come from the direct discharge of sewage and also could be enriched in the polluted aquatic environment. Sewage carried pathogenic bacteria and amounts of nutrients, strongly impacting the original river aquatic ecosystem. The polluted river water and sediment also contributed to the growth of pathogenic bacteria carried by the sewage and originally present in the river, which has been proved in the incubation experiments.

### Self-purification of river decreased pathogenic bacteria abundance

After the raw sewage entered the river, the self-purification influenced the river's environmental conditions and then affected the further migration and deposition of pathogenic bacteria. The content of COD, NH_4_^+^, and TP gradually decreased from Site-2 to Site-4. The reason behind the improvement in water quality from site-2 to Site-3 is mostly due to river self-purification, which may entail involve physical, chemical, or biological processes that contribute to the removal of pollutants from the river^50^. Furthermore, Site-4 has superior water quality over Site-2 and 3. This might be because the treated wastewater released from STP prior to Site-4 had fewer pollutants than Site-3, which in turn diluted the water and enhanced its quality. With the pollutant concentrations decreased, the abundance of genes related to *Vibrio cholerae, Staphylococcus aureus,* and *Helicobacter pylori* decreased and the diversity and richness of microbial community structure also recovered over time. The response of the ecosystem to chemically-driven environmental changes is modulated by physical and biological phenomena. Self-purification could keep the maintenance or restoration of river ecology and influencing factors may include seasonal fluctuations, the microbiological community present^42^, and temperature^51^. The biological processes in river ecosystem are mainly carried out by endogenous and exogenous microorganisms that can remove organic and inorganic pollutants in contaminated water bodies, essential for maintaining a stable ecosystem dynamics state, in terms of function and structure^51,52^. Another concern is that microbes could be shielded by physical embedding in organic matter, suspended particles, and occlusion of a biofilm, making them less susceptible to the inactivation action of disinfectants^50^. Most pathogens, which came from raw sewage, could also be absorbed by animals, plants, and sediments in the river. Therefore, it is urgent to further focus on the migration and transformation mechanism of pathogenic bacteria in sediments. Although the microbial structure was slightly restored and the abundance of most pathogenic bacteria was also slightly reduced through river self-purification, direct discharge of raw sewage caused severe and short-term irreversible damage to the river environment.

### Significance of this research

This study contributes to an improved understanding of the effects of sewage discharge on the changes of microbial communities and the abundance of pathogenic bacteria in river sediments. Despite water and sewage treatment technologies continue to achieve rapid progress in recent years, waterborne pollution remains a major threat to public health worldwide^53^. This paper brings to the fore the need for comprehensive research into the movement and behavior of these microorganisms in river sediment after raw sewage is contaminated. And besides, with the evidence of increased pathogen abundance, we are here to demonstrate and highlight the risks of direct sewage discharge. Wastewater as a secondary source of transmission is not given full attention, especially in third-world countries. If wastewaters act as the source of transmission, it will be hard to break the chain of pathogen transmission in the third world countries and the consequence will be faced by developed nations as well. There is a common practice to discharge wastewater directly into rivers, canals, and lakes without any treatment in third-world countries. The wastewater carried pathogens can go to a big population of the world and create population crises in the world. Therefore, measures should be taken as soon as possible in third-world countries to break the chain of pathogen transmission. Strict surveillance of wastewater treatment is suggested to stop the spread of pathogens in the human community.

## Conclusion

Here, the effect of raw sewage discharge on the river system was studied. Direct discharge brought high concentrations of pollutants to the river, especially COD and nitrogen fractions. The deterioration of water quality had a serious impact on the surface mud microbial community of the river, and microbial diversity and abundance were reduced. The relative abundance of nitrogen metabolizing functional microorganisms and pathogenic bacteria increased after the direct discharge of sewage. Pathogens in the Daxin River could come from the direct discharge of sewage and also could be enriched in the polluted aquatic environment. As the river flowed, the water quality and microbial community structure of the river gradually recovered, and the abundance of pathogenic bacteria continued to decline, showing the self-purification ability of rivers. Although self-purification of rivers can restore a certain microbial diversity, the sewage direct discharge still causes irreversible effects, and river ecosystems cannot be restored to their original state. In conclusion, direct discharge of wastewater should be forbidden with regard to the negative impact on entering the ecosystem, especially on the lives that depend on the ecosystem for sustenance.

## Data Availability

Data supporting the results of this study are available from the corresponding author upon reasonable request. The sequence of the 16S rDNA obtained in this study has been submitted to the NCBI Sequence Reading Archive (SRA) with the accession numbers SUB9796686:PRJNA754023.
